# MRI-Based Assessment of Safe Margins in Tumor Surgery

**DOI:** 10.1155/2014/686790

**Published:** 2014-02-20

**Authors:** Laura Bellanova, Thomas Schubert, Olivier Cartiaux, Frédéric Lecouvet, Christine Galant, Xavier Banse, Pierre-Louis Docquier

**Affiliations:** ^1^Computer Assisted and Robotic Surgery (CARS), Institut de Recherche Expérimentale et Clinique (IREC), Université catholique de Louvain Tour Pasteur +4, Avenue Mounier, 53, 1200 Brussels, Belgium; ^2^Département D'imagerie Médicale, Cliniques Universitaires Saint-Luc 10, Avenue Hippocrate, 1200 Brussels, Belgium; ^3^Département de Pathologie, Cliniques Universitaires Saint-Luc 10, Avenue Hippocrate, 1200 Brussels, Belgium

## Abstract

*Introduction*. In surgical oncology, histological analysis of excised tumor specimen is the conventional method to assess the safety of the resection margins. We tested the feasibility of using MRI to assess the resection margins of freshly explanted tumor specimens in rats. *Materials and Methods*. Fourteen specimen of sarcoma were resected in rats and analysed both with MRI and histologically. Slicing of the specimen was identical for the two methods and corresponding slices were paired. 498 margins were measured in length and classified using the UICC classification (R0, R1, and R2). *Results*. The mean difference between the 498 margins measured both with histology and MRI was 0.3 mm (SD 1.0 mm). The agreement interval of the two measurement methods was [−1.7 mm; 2.2 mm]. In terms of the UICC classification, a strict correlation was observed between MRI- and histology-based classifications (*κ* = 0.84, *P* < 0.05). *Discussion*. This experimental study showed the feasibility to use MRI images of excised tumor specimen to assess the resection margins with the same degree of accuracy as the conventional histopathological analysis. When completed, MRI acquisition of resected tumors may alert the surgeon in case of inadequate margin and help advantageously the histopathological analysis.

## 1. Introduction

Limb-salvage surgery is nowadays the ideal treatment for bone and soft tissue sarcoma [1]. Although histological grade and tumor size are important prognostic factors, inadequate resection margins remain one of the most significant predictors of local recurrence for bone and soft tissue sarcomas, even in the presence of adjuvant therapies [2–4]. A local recurrence usually impairs limb preservation and functional outcomes, but it is also correlated with an increased risk of metastatic disease development [5, 6]. Identified causes of local recurrence are insufficient resection margins, undetected metastasis, for instance in the lymph nodes, and tumor venous emboli. While other causes cannot be treated by surgery alone and require adjuvant treatment, insufficient resection margins can be avoided with a careful dissection and safe resection margins [7, 8]. 

Gross extemporaneous macroscopical analysis of the excised tumor specimen by the surgeon, followed by delayed histopathological analysis, is the conventional method to evaluate the safety of the resection margins. In a histopathological study, Picci et al. correlated local recurrence with insufficient resection margins [9]. Histologic assessment of margin status was shown useful for predicting local recurrence of cutaneous malignant tumors in dogs and cats treated by means of excision alone [10]. Several prognostic classifications have been published to histologically evaluate surgical margins and identify high-/low-risk groups for local recurrence after limb salvage surgery. A standardized classification was created by the Union for International Cancer Control (UICC). It distinguishes R0 as *in sano* resection R1 as possible microscopic residuals (margin between 0 and 1 mm) and R2 as macroscopic residual disease [11].

Magnetic resonance imaging (MRI) is widely used for oncological diagnosis, disease extension assessment, surgical planning, and postoperative followup [12]. Current available resolution of preoperative MRI images enables accurate delineation of the tumor boundaries for surgical planning purposes [8, 13]. However, MRI has never been considered as a possible tool for assessing the safety of resection margins on the freshly explanted tumor specimen.

This experimental study, performed on rodent models, aimed at investigating the feasibility of using MRI to assess the resection margins of excised tumor specimen using the UICC classification and comparing MRI with the conventional extemporaneous histopathological method.

## 2. Materials and Methods

### 2.1. Animals and Tumor Induction

Experiments on animals were carried out in compliance with the Institutional Ethics Committee for Laboratory Animal Experimentation (CE Accred. number UCL/MD/2011/022). Animals were housed according to the guidelines of the Belgian Ministry of Agriculture and Animal Care. Seven 9-week-old male WAG/RijHsd rats (Harlan Laboratories, Boxmeer, The Netherlands) were used as recipients. Fragments from a syngeneic rhabdomyosarcoma [14] of approximately 1 mm^3^ were grafted intramuscularly in both thighs under general anesthesia by Isoflurane inhalation (Forene, Abbott, Diegem, Belgium). The sarcoma was implanted bilaterally in the pelvic region/proximal thigh. Implanted tumor fragments grew within a 3- to 4-week delay to reach a gross volume of approximately 2 to 3 cm^3^ at the time of imaging. No rat died nor showed any significant impairment in general status during the period of tumor growth.

Three to four weeks postimplantation, the rats were sacrificed by T61 (Intervet International GmbH, Germany) intracardiac injection under general anesthesia. In four rats (Rat1, Rat2, Rat3, and Rat4), the two tumors were explanted separately resulting in two resection specimens per rat. A wood stick was inserted in each specimen and considered as reference axis for the comparison between MRI-based and histological margin evaluation. For the three remaining rats (Rat5, Rat6, Rat7), “en-bloc” resection of the pelvis with both femurs was performed and the axis of the spine was used as reference axis during the evaluation process.

### 2.2. MRI Acquisition

MRI of the specimens was done after resection. The images were acquired using a 1.5-Tesla MRI unit (Gyroscan NT Intera T15; Philips Medical Systems, Bets, The Netherlands) with contiguous slices. Reference axis for slicing was the wood stick for the specimens excised from Rat1, Rat2, Rat3, and Rat4, and the spine axis for the specimens excised from Rat5, Rat6, and Rat7. The acquisition parameters used for the specimens were specified as follows: reconstruction matrix 176 × 176, repetition time 1500 ms, echo time 15 ms, section thickness 0.5 mm, and spacing between slices 0.5 mm. The specimens were scanned and the sequences saved in DICOM format prior to analysis with the picture archiving and communication system (PACS, Carestream, Health, NY, USA). The distance measurement tool of the PACS system was used to measure the resection margins as the distance (in mm) between the tumor boundary and the specimen edge.

### 2.3. Histology

After MRI acquisition, each excised specimen was fixed in methanol and methylmethacrylate (MMA) embedded. The polymerized blocks were sliced with 200-*μ*m thickness with a diamond band saw (EXAKT, D-22581, Norderstedt, Germany) perpendicular to the same reference axis used in MRI slices (wood stick for the specimens excised from Rat1, Rat2, Rat3, and Rat4, and spine axis for the specimens excised from Rat5, Rat6, and Rat7). The slices were ground and polished up to 120 *μ*m between two sheets of ground glass, stained with 1% Methylen Blue (85662, MERCK, Hohenbrunn, Germany) and 1% Fuchsin (B-2340, Sigma Aldrich, St-Louis, USA), and finally mounted on glass.

The histological slices were scanned with a flatbed scanner (Scanner Canon LiDE 210, Diegem, Belgium) and images were saved in “jpg” format. Evaluation of the resection margins was performed using ImageJ 1.43i software (ImageJ, Image Processing and Analysis in JAVA, National Institute of Health, Bethesda, MD, USA) and the built-in distance measurement tool.

### 2.4. Evaluation

**Figure 1:**
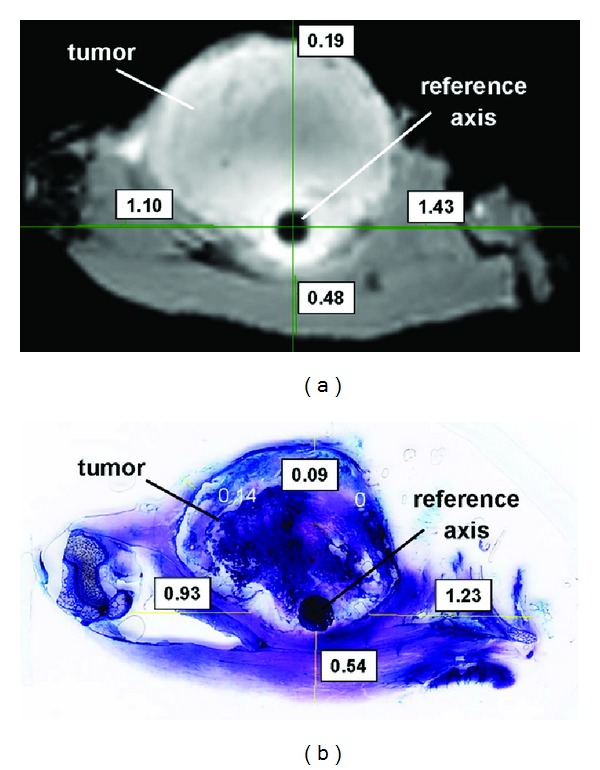
Example of a histological slice (b) associated with its corresponding MRI slice (a) of the same excised tumor specimen for the comparison of the two measurement methods. In this case, the reference axis for the measurements is the wood stick inserted in the excised specimen. Resection margins are measured in mm along the straight lines drawn manually by the operator.

Each histological slice was associated with its corresponding MRI slice (Figure 1). Eighty-six pairs of corresponding MRI and histological slices were available for the comparison between MRI-based and histological margin evaluation. On each pair of slices, the operator manually drew straight lines from the reference axis (wood stick or spine axis) to the slice boundary (Figure 1). Resection margins were measured in mm along the straight lines between the tumor boundary and the specimen boundary. In total, 498 measurements were performed on the histological slices and compared with the corresponding measurements on the MRI slices. The two series of 498 measured resection margins were also classified using the UICC classification as R0 (*in sano* if more than 1 mm), R1 (possible microscopic residuals if between 0 and 1 mm), and R2 (macroscopic residuals if 0).

### 2.5. Statistics

**Figure 2:**
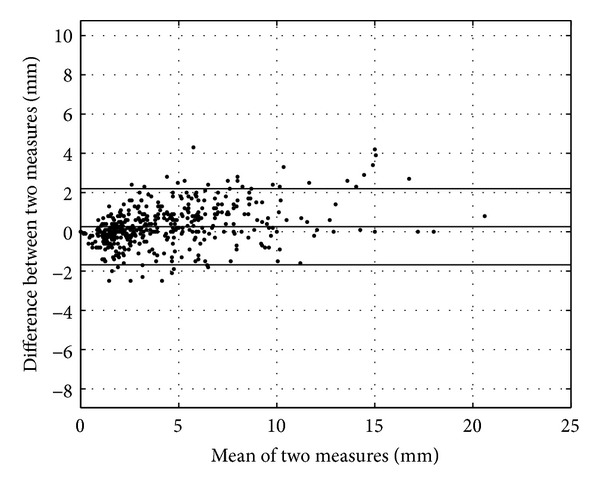
Bland and Altman plot of the two measurement methods (histology and MRI). The dashed line represents the mean value of the differences between the two measurement methods, and the lines above and below it represent the agreement interval.

Statistical analyses were performed with PASW 19 (formerly SPSS, IBM, New York, NY, USA). The one-sample Kolmogorov-Smirnov test and Q-Q plots were used to assess normality of values. A Bland and Altman plot (or difference plot) [15] was used to compare the two methods (MRI and histology) (Figure 2). In this plot, the differences between the two methods were plotted against the averages of the two methods. Horizontal lines were drawn at the mean difference and the limits of agreement (mean difference ±1.96 times standard deviation). The Pearson correlation was used to assess the linear relationship between the two methods. Statistical differences between groups were tested by Student's paired *t*-tests. A *P* value < 0.05 was considered significant. Results were expressed as mean and standard deviation (SD).

## 3. Results

**Figure 3:**
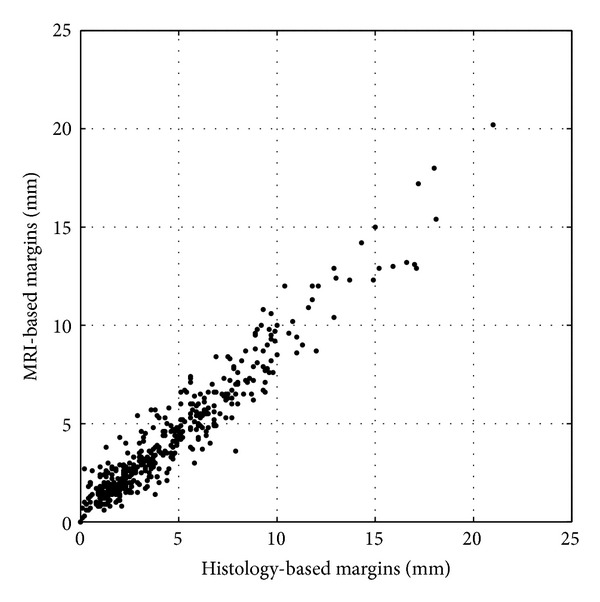
Correlation between the two measurement methods.

The mean difference between the 498 margins measured both with histology and MRI was 0.3 mm (SD 1.0 mm) (Figure 2). Agreement interval of the two measurement methods was (−1.7 mm; 2.2 mm). A scatter plot showed the correlation between the two methods (Figure 3). The Pearson correlation coefficient was 0.97.

In terms of the UICC classification, 95.4% (475/498) of the resection margins were classified similarly by the two measurement methods (Table 1). In 3.2% (16/498), the resection margins were classified R1 using the histological measurements and R0 using the MRI measurements. On the contrary, 1.4% (7/498) were classified R1 with the MRI measurements and R0 with the histological measurements. R2 classification was fully identical with both methods. A strict correlation was observed between MRI- and histology-based classifications (*κ* = 0.84, *P* < 0.05).

## 4. Discussion

This experimental study showed the feasibility to use MRI imaging of excised tumor specimen to assess the resection margins with the same degree of accuracy than the conventional extemporaneous histopathological analysis. The Bland and Altman plot showed that the two measurement methods were concordant. Moreover, a good correlation coefficient was found. In terms of the UICC classification, good kappa coefficient was found, meaning a strict correlation. All cases with macroscopic residuals (R2) were concordant with the two methods and none was misinterpreted as R1 by one of the method. The only differing interpretations were between R0 and R1.

Magnetic resonance imaging remains the most accurate noninvasive tool for staging bone and soft-tissue extent of musculoskeletal sarcomas as it displays precisely the tumor, the compartmental spread, and the neurovascular and articular involvement [14]. Technological advances over the last years allowed significant improvement in tumor delineation and extension [15]. When the tumoral specimen is resected, it is available for MRI acquisition (in 30 minutes). Result of the MRI can be analysed by the surgeon before the end of the surgical procedure, allowing if needed further resection in case of inadequate margin. Nevertheless, MRI may not replace the anatomopathological specimen analysis as it gives no information about the local effect of chemotherapy, as expressed by percentage of tumor necrosis. The result of the thorough examination of the tumor specimen and of its resection margins will usually be available within the week.

Extemporaneous thorough examination of the entire specimen by the pathologist is not possible. MRI imaging of the entire specimen could on the contrary be quickly obtained and analysed by the surgeon or a radiologist and lead to immediate further resection. Serial histology of the specimen perpendicularly to the spine/marker allowed a good match between the MRI slices and the histological slides. A statistically significant high correlation was found between the anatomopathological and MRI findings. In 49.8% of the measures, larger resection margins were secondarily diagnosed by histology. This finding demonstrates the safety of the procedure. Only in a few cases, histologically R1 resections were found R0 by MRI (3.2%; 16/498).

MRI could also be used as additional tool to help the pathologist, by a preliminary investigation, to focus his attention on doubtful areas. This could be very effective in case of voluminous tumors where pathologic study of the whole resection specimen is difficult and time consuming.

One of the limitations of the MRI evaluation is the low resolution at the edge of the specimen. Additional studies have to improve the MRI analysis of the tumoral contour. MRI acquisition with the specimen in a formalin bath could increase the resolution at the specimen border. Moreover, extensive clinical trials in which both MRI and anatomopathology are undertaken together should be realized before one could however consider abandoning extemporaneous examination.

Another limitation of this study is the relatively small size of the resection specimens (rat limb). Clinical human specimens are generally larger and could be more difficult or complex to interpret.

Complementary animal and in vivo studies should be performed to fully validate the observed results in terms of accuracy, repeatability, and time.

## 5. Conclusion

When completed, intraoperative MRI acquisition of resected tumors may enable immediate assessment of surgical margins and help advantageously the histopathological analysis.

## Figures and Tables

**Table 1:** Cross tabulation of MRI-based classification by histology-based classification of the 498 measured resection margins.

Histology-based classification	MRI-based classification		
	R0	R1	R2
R0	404	7	0
R1	16	10	0
R2	0	0	61
